# Memory and Representation of Vision-Related Verbs in Early Blind Individuals

**DOI:** 10.5334/joc.458

**Published:** 2025-08-22

**Authors:** Léo Dutriaux, Roberto Bottini

**Affiliations:** 1 UniversitéLumière Lyon 2, Laboratoire d’Études des Mécanismes Cognitifs, 69007 Lyon, France; 2 Center for Mind/Brain Sciences, University of Trento, 38122, Trento, Italy

**Keywords:** Grounded cognition, Long Term Memory, Blindness, Concepts, Language

## Abstract

Theories of Embodied and grounded cognition posit that knowledge retrieval is rooted in sensorimotor simulations of past experiences. Accordingly, individuals with diverse sensorimotor experiences may retrieve knowledge differently. Here, we asked whether and how congenital blind individuals remap the representation of vision-related verbs in the motor system. Participants memorized lists of phrases combining an object to an action-related (“to take a guitar”), vision-related (“to see a guitar”), or control verb (“to hear a guitar”). The lists were either learned with the hands at rest or behind their back. Results replicated previous findings showing that recall for action-related phrases was lower in both groups when they were learned with the hands behind the back. As expected, posture impacted the memory of vision-related phrases only in blind people, although in the opposite direction. These findings provide evidence for the sensorimotor grounding of knowledge and shed light on how blind individuals represent knowledge.

## Introduction

In contrast to traditional views that treat cognition as abstract and amodal, theories of embodied and grounded cognition claim that cognitive processes are deeply rooted in our bodily states, actions, and interactions with the environment. These theories (e.g., Barsalou, 1999; Versace et al., 2014) propose that knowledge is grounded in sensorimotor simulations, and that these simulations are embodied in the sense that they are shaped by sensorimotor interactions, and consequently by the physical constraints of the individual’s body. Evidence supporting these theories indicates that the processing of action-related language activates the motor cortex (e.g., Tettamanti et al., 2005) and facilitates actions that are compatible with its meaning (e.g., Scorolli & Borghi, 2007). Similarly, processing language related to visual properties like color or movement activates corresponding visual areas (e.g., Saygin, McCullough, Alac, & Emmorey, 2010; Simmons et al., 2007; Wallentin et al., 2011) and impacts behavioral responses related to color or movement (e.g., Meteyard, Bahrami, & Vigliocco, 2007; Richter & Zwaan, 2009).

However, some have raised concerns that much of this evidence is correlational, and it remains unclear whether these effects reflect sensorimotor activity that is functionally relevant to the representation of knowledge (Mahon & Caramazza, 2008; Ostarek & Bottini, 2021). Indeed, sensorimotor simulation might either be strictly necessary and functionally relevant, not strictly necessary but functionally relevant, or neither necessary nor functionally relevant. Ostarek and Bottini (2021) described three types of causal experiments to investigate this question: (1) studying how congenital sensory-motor disorders, (2) acquired sensory-motor disorders, or (3) behavioral sensorimotor interferences impact conceptual processing. Specifically, congenital sensory-motor disorders constitute an ideal test bed for testing this question. Indeed, if knowledge is grounded in embodied sensorimotor experience, the lack of a sensorimotor modality should entail a different experience of the world, and consequently a different grounding of knowledge (Casasanto, 2009). In this context, understanding how individuals with congenital blindness represent vision-related knowledge will provide decisive evidence regarding the functional role of sensorimotor systems in knowledge representation (see also Vannuscorps et al., 2014; Vannuscorps & Caramazza, 2019 for similar ideas with congenital limb aplasia).

Because the largest effect of the absence of vision should arise with concepts whose referents can only be experienced through vision, color knowledge has received much attention. For example, studies have shown that blind individuals often struggle to name the color of animals (Kim et al., 2019), and that color implicitly structures the category of fruits and vegetables in sighted but not in blind participants (Connolly et al., 2007). However, it is important to note that in these studies, the researchers mentioned that their congenitally blind participants all developed color knowledge to some extent, sometimes even on par with the color knowledge of sighted participants. Additionally, it has been demonstrated that knowledge about color similarity and color properties is largely similar in both groups (Barilari et al., 2018; Kim et al., 2019, 2021; Saysani et al., 2018). Nevertheless, the absence of vision does seem to impact the development of color knowledge in blind individuals. This difference might arise because the color knowledge acquired through language and categorical inference (Kim et al., 2019; Ostarek et al., 2019) does not always provide sufficient information to compensate for the lack of visual information. Two recent neuroimaging studies are consistent with this interpretation, showing that color similarity is encoded in the visual cortex region responsible for color processing (V4 regions) only in sighted participants, while color similarity coding in blind individuals was found only in regions associated with abstract representations (Bottini et al., 2020; Wang et al., 2020). Although correlational, This finding is in line with the idea that color knowledge in sighted people relies on visual information, whereas in blind people, it relies on abstract representations acquired through language and inference.

For remappable concepts and properties, the expected pattern of results is different. These concepts are called remappable in the sense that they are usually acquired through vision but can also be acquired through other sensorimotor modalities (e.g., size and shape). Therefore, the same property can be remapped from one sensorimotor modality to another. For instance, for shape, while the visual system is preponderant for sighted people, the haptic and motor systems should compensate for blind people because they can perceive shapes through tactile exploration. However, several neuroimaging studies seem to provide evidence against this hypothesis (Bi et al., 2016; Mattioni et al., 2020; Peelen & Downing, 2017). They showed that areas known to be involved in visual processing in blind people are also involved in the representation of knowledge in both sighted and blind. For example, imagined spatial navigation as well as the processing of words related to large objects activated the parahippocampal place area, occipital place area, and retrosplenial cortex, all known to be involved in visual scene processing (He et al., 2013; Sigismondi et al., 2024). Similarly, concepts of manipulable objects activated the lateral temporal-occipital complex, which is usually observed during the vision of tool or tool-related actions (Peelen et al., 2013), and objects’ shape is represented in the inferolateral occipitotemporal cortex in both sighted and blind (Xu et al., 2023). Finally, action verbs activated in both groups the left posterior middle temporal gyrus, known for its involvement in the processing of visual movements (Bedny et al., 2012; Bottini et al., 2020; Noppeney et al., 2003). Importantly, in these studies, there were no differences between blind and sighted participants in modality-specific areas. This indicates that the brain network related to conceptual processing is not impacted by congenital blindness and that these putative visual areas encode abstract information instead of modality-specific information. However, even if these fMRI experiment studied congenitally blind people, these studies remain correlational since it is unknown whether these activations in the visual system are causally involved in the processing of vision-related concepts. Furthermore, it is known that the visual areas of early blind individuals can be recycled for other perceptual modalities (Dormal & Collignon, 2011; Müller et al., 2019) and might yet preserve their functional role (Reich et al., 2011; Ricciardi et al., 2007). It is then possible that the areas supporting visual simulation of knowledge in sighted people support haptic or auditory simulations of the same knowledge in blind people. Nonetheless, behavioral studies showed that blind individuals have similar knowledge to sighted individuals regarding remappable knowledge. Early work showed, for instance, that from an early age, children with congenital blindness understand and use appropriately vision verbs like *look* or *watch* (Landau & Gleitman, 1995). A recent study on adults consistently showed that similarity judgments of such vision verbs resulted in seemingly identical ratings in sighted and blind participants (Kim et al., 2019). Blind individuals also understand novel visual metaphors just as well as sighted participants (Minervino et al., 2018). One problem with these behavioral studies is that they evaluate explicit knowledge related to remappable knowledge, but they cannot tell much about the type of information involved in this knowledge. Indeed, it is possible that other sensorimotor modalities are enough to compensate entirely for the lack of vision to represent remappable knowledge. One possibility to causally assess whether vision-related knowledge is remapped onto non-visual modalities in early blind individuals is through the third causal paradigm suggested by Ostarek and Bottini (2021): sensorimotor interference paradigms. The present study aims to test this using a motor interference paradigm and vision-related verbs. To this end, this work draws on a previous study (Dutriaux et al., 2019) where sighted participants learned lists of manual action-related phrases (e.g., “to take a cup”) and vision-related phrases (e.g., “to see a cup”). Crucially, during the learning phase, participants kept their hands either at rest in front of them or behind their back. Results showed that keeping the hand behind the back had a detrimental effect on the memory of action phrases, but not on the memory of vision phrases. This effect presumably arose because the body is not available to act when the hands are behind the back, which inhibits the motor simulation related to action phrases and reduces the strength of the resulting memory trace. Neuroimaging studies later backed up this interpretation by demonstrating that motor activity is involved in this effect (de Vega et al., 2021; Onishi et al., 2022). The present experiment aimed to use a similar paradigm comparing the memory of action, vision, and control phrases in sighted and early blind participants. The main hypothesis concerns vision phrases. Indeed, since blind people “see” the world through manual exploration of the environment, the meaning of such verbs should rely in part on the motor system. Thus, we expected a detrimental effect of the hands behind the back on the memory of vision phrases in blind but not sighted participants. Alternatively, if the meaning of vision phrases does not remap on the motor system, there should be no postural effect on their memory. A secondary hypothesis was that the hands behind the back should have a more substantial effect on the memory of action phrases in blind than in sighted participants. Indeed, the representation of these verbs should rely even more on motoric information in the absence of visual information. Finally, no effect of posture was expected with control verbs since they were neither vision nor motor-related.

## Method

### Participants

Fifty participants participated in this experiment: 25 early-blind individuals (EB) and 25 sighted control participants (SC). Participants were matched pairwise (see Table 1) for sex (13 females and 12 males in each group), age (EB: mean = 35.44, SD = 7.38; SC: mean = 34.92, SD = 8.88), and years of education (EB: mean = 15.16, SD = 3.00; SC: mean = 15.16, SD = 3.00). There were 24 right-handed and one left-handed participants in each group. Nineteen of our blind participants lost sight at birth, while the remaining 6 participants lost it before four years old. None of them reported having visual memories. All participants were blindfolded during the task. Finally, they all gave informed consent to the experimental procedure and received a financial reimbursement of 8 Euros.

**Table 1:** Demographic information of the early blind and their matched sighted control (M = male; F = female; EB = early blind; SC = sighted control; M = mean; SD = standard deviation).


EARLY BLIND	AGE	GENDER	YEARS OF EDUCATION	ONSET OF BLINDNESS	ETIOLOGY	SIGHTED CONTROLS	AGE	GENDER	YEARS OF EDUCATION

EB01	30	M	18	3 years	Glaucoma	SC01	28	M	18

EB02	34	M	13	Birth	Congenital anophthalmia	SC02	34	M	8

EB03	33	F	18	Birth	Retinopathy of Prematurity	SC03	29	F	18

EB04	40	M	13	7 months	Retinopathy of Prematurity	SC04	37	M	13

EB05	29	F	18	Birth	Congenital microphthalmia	SC05	29	F	18

EB06	30	F	18	Birth	Agenesis of the optic nerves	SC06	27	F	18

EB07	31	F	13	Birth	Retinitis pigmentosa	SC07	30	F	13

EB08	55	F	13	1 year	Retinoblastoma	SC08	55	F	13

EB09	28	M	13	Birth	Congenital toxoplasmosis	SC09	32	M	13

EB10	33	M	13	Birth	Retinal detachment	SC10	30	M	13

EB11	34	F	13	Birth	Retinopathy of Prematurity	SC11	37	F	13

EB12	30	M	18	Birth	Retinopathy of Prematurity	SC12	28	M	18

EB13	38	F	13	Birth	Congenital cataracts	SC13	41	F	13

EB14	30	F	18	Birth	Retinopathy of Prematurity	SC14	27	F	18

EB15	32	F	13	1.5 years	Retinoblastoma	SC15	28	F	13

EB16	35	F	16	4 years	Retinitis pigmentosa	SC16	36	F	16

EB17	39	F	18	Birth	Retrolental fibroplasia	SC17	41	F	18

EB18	34	M	13	Birth	Retinitis pigmentosa	SC18	33	M	13

EB19	30	F	18	Birth	Retinopathy of Prematurity	SC19	27	F	18

EB20	36	M	16	Birth	Optic nerve hypoplasia	SC20	35	M	16

EB21	27	M	13	3 years	Dominant optic atrophy	SC21	30	M	13

EB22	43	M	21	Birth	Congenital retinopathy	SC22	47	M	21

EB23	34	F	18	Birth	Retinopathy of Prematurity	SC23	28	F	18

EB24	52	M	8	Birth	Congenital glaucoma	SC24	59	M	13

EB25	49	M	13	Birth	Optic nerve hypoplasia	SC25	45	M	13

*M*	35.44		15.16				34.92		15.16

*SD*	7.23		2.94				8.60		2.94


### Stimuli

In this experiment, we used 72 Italian object names and 36 Italian verbs, including 12 manual action-related verbs, 12 vision-related verbs, and 12 control verbs. Objects and verbs were combined to form phrases in the structure of verb + article + noun. Three sets of 72 sentences were built, such as each object was associated with all three verb types across participants. For instance, *diary* was associated with the verb *to open* in set 1 (e.g., *aprire un diario*/to open a diary), with *to see* in set 2 (e.g., *vedere un diario*/to see a diary), and *to lose* in set 3 (e.g., *perdere un diario*/to lose a diary). Each of the 36 verbs was paired with two different objects across each set of phrases (see the Appendix for the complete list of stimuli).

Verbs did not differ across verb types in terms of frequency (Zipf scale (Crepaldi et al., 2015), action: *M* = 45.41, *SD* = 1.17; vision: *M* = 44.68, *SD* = 1.14; control: *M* = 45.22, *SD* = 1.17; Kruskal-Wallis *H*(2) = 0.09, *p* = .956), letters number (action: *M* = 8.83, *SD* = 1.94; vision: *M* = 7.67, *SD* = 1.87; control: *M* = 7.91, *SD* = 1.83; *H*(2) = 2.22, *p* = .329), or auditory length (action: *M* = 718 ms, *SD* = 112 ms; vision: *M* = 766 ms, *SD* = 141 ms; control: *M* = 662 ms, *SD* = 157 ms; *H*(2) = 3.56, *p* = .169). We generated auditory files for the stimuli using the female voice of the IBM Watson Text-to-Speech software.

### Design and procedure

Participants were assigned randomly to one of the three sets of 72 phrases. The 72 phrases were divided into eight lists, each containing three sentences of each type with no verb repetition. A filler phrase with the same syntactic structure was added to the beginning of all lists to control for primacy effects and was not included in the analyses. The order of presentation of the lists as well as the order of the phrases within the lists (except the filler phrase, which was always first) were randomized across participants. In total, participants were presented with eight lists of ten phrases, including one filler sentence, three action phrases, three vision phrases, and three control phrases.

At the start of the experiment, all participants were blindfolded and asked to listen to the auditory instructions. They were then presented with an additional list of five sentences as training. For each list, participants underwent three phases: 1) a learning phase, 2) a distractive task, and 3) a cued recall task. All tasks were presented auditorily using a headset and programmed in Python 3 using the Neuropsydia package (Makowski & Dutriaux, 2017).

In the learning phase, participants were asked to memorize the phrases while adopting either of two experimental postures: keeping their hands on the table in a relaxed posture (front posture) or holding one wrist with the other hand behind their back (back posture). Twelve participants in each group performed the learning phase of the first four lists in the front posture and the four last in the back posture, while the remaining 13 participants of each group started with the back posture and ended with the back posture. During this phase, phrases were presented one after the other (duration range = 1066–2233 ms) with a stimulus onset asynchrony (SOA) of 4000 ms.

The distractive task lasted about one minute and consisted of a simple auditory discrimination task between pairs of tones. For each of the 20 trials, participants kept their hands on the table and were asked to orally indicate whether the first tone, second tone, or neither had a higher pitch. The duration of each tone was 300 ms, and the inter-stimulus interval within pairs of tones was 400 ms. Participants were given 2000 ms to respond. This task aimed to avoid recency effects in memory and was not analyzed further.

In the cued recall task, the verbs of the previously presented list were presented one after the other in a random order (duration range = 465–994 ms), with an SOA of 5500 ms. Still with their hands on the table, participants were instructed to recall the object associated with each verb orally. The experimenter recorded the responses to measure accuracy in a later step.

## Results

Data analyses were performed using *R* (version 4.4.0). Mixed models were computed using the *lme4* package (version 1.1-35.3; Bates et al., 2015), p values were obtained computing Wald Chi-square tests using the *car* package (version 3.1-2), and pairwise post-hoc contrasts were computed using the *emmeans* package (version 1.10.2; Lenth, 2022). Data were plotted using the package *ggplot2* (version 3.5.1; Wickham, 2016).

**Figure 1:**
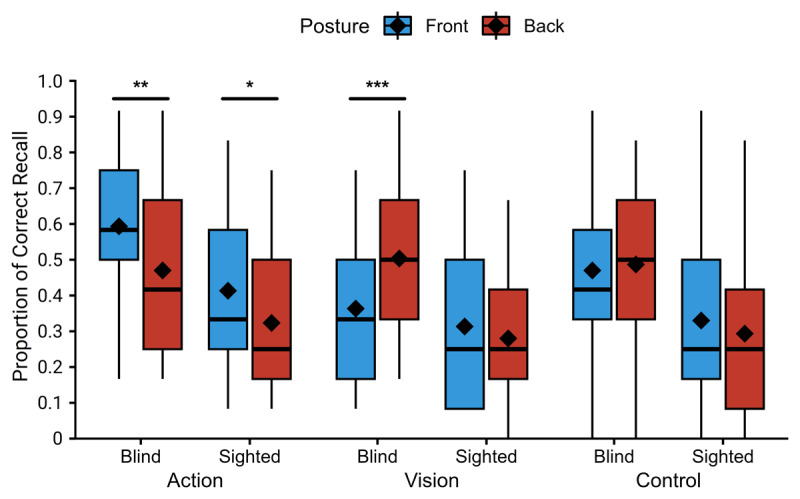
Percentage of recalled objects as a function of posture, group, and phrase type. Diamonds represent the mean; box and whisker plots represent the median and inter-quartile range (* *p* < .05; ** *p* < .01; *** *p* < .001).

First, the minimum accuracy at the distractive task was 79%, with an average accuracy of 90% for sighted participants and 95% for early blind participants. This indicates that all participants were distracted by this task. Figure 1 shows the mean proportion of words correctly recalled. They were analysed using a general linear mixed model on recall accuracy including group, phrase type, and posture as fixed effect; and participants and items as random effect. The relevant random slopes were added to respect the maximum random effects structure (Barr et al., 2013). That is, random slopes for all interactions and main effects were added for items, while only random slopes of the interaction between phrase type and posture and their main effect were added for participants (since group is a between-participant variable).

Results showed a reliable main effect of group (χ^2^(1) = 10.61, *p* = .001), where accuracy was higher for blind than sighted participants. There was also a reliable main effect of posture (χ^2^(1) = 6.58, *p* = .01), with better performance with the front posture than with the back posture. Then, there was also a main effect of phrase type (χ^2^(2) = 24.81, *p* < .001). Post-hoc analyses showed that this main effect was driven by the difference in accuracy between action phrases and vision phrases (Odds Ratio (OR) = 1.61; z = 2.66; *p* = .021; action vs. abstract: OR = 1.36; z = 1.89; *p* = .142; vision vs. abstract: OR = 0.84; z = –1.09; *p* = .520). Turning now to interactions, there was a significant two-way interaction between group and phrase type (χ^2^(2) = 6.09, *p* = .048) and between posture and phrase type (χ^2^(2) = 14.51, *p* < .001). Crucially, the predicted three-way interaction between group, phrase type, and posture was also significant (χ^2^(2) = 6.00, *p <* .05).

To understand all these interactions further, we performed three separate analyses using one general mixed model for each phrase type. They had a similar structure to the full model, only removing phrase type from the models. Concerning the effect of group for each phrase type, better performance for blind participants was observed only for action and control phrases (action: χ^2^(1) = 10.39, *p* = .001, control: χ^2^(1) = 4.64, *p* = .031), but not for visual phrases (χ^2^(1) = 1.02, *p* = .313). Analyses of the effect of posture for each phrase type showed that the main effect of posture that was observed in the full model was not interpretable. Indeed, while recall accuracy was worse with the back posture for action phrases (χ^2^(1) = 8.20, *p* = .004), it was better with the back posture for vision phrases (χ^2^(1) = 10.19, *p* = .001), and there was no reliable effect of posture for control phrases (χ^2^(1) = 0.00, *p* = .987).

Concerning the group × posture interaction, the pattern of results differed across phrase types as well. First, as expected, the interaction was not significant in control phrases (χ^2^(1) = 0.57, *p* = .450), posture having no reliable effect on the recall of control phrases in either group (blind: OR = 1.00; z = 0.02; *p* = .987; sighted: OR = 1.26; z = 1.05; *p* = .296). Second, contrary to our hypothesis, this interaction was not significant in action phrases either (χ^2^(1) = 0.27, *p* = .602), as a comparable detrimental effect of the front posture was observed in both groups (blind: OR = 1.81; z = 2.86; *p* = .004; sighted: OR = 1.55; z = 2.00; *p* = .045). Finally, results showed, as hypothesized, a reliable group × posture interaction (χ^2^(1) = 9.14, *p* = .003), with an effect of posture on the recall of vision phrases only in blind participants. Surprisingly, however, blind participants were better at recalling vision phrases when they learned them in the back posture than in the front posture (blind: OR = 0.53; z = –3.19; *p* = .001; sighted: OR = 1.37; z = 1.35; *p* = .178).

**Figure 2:**
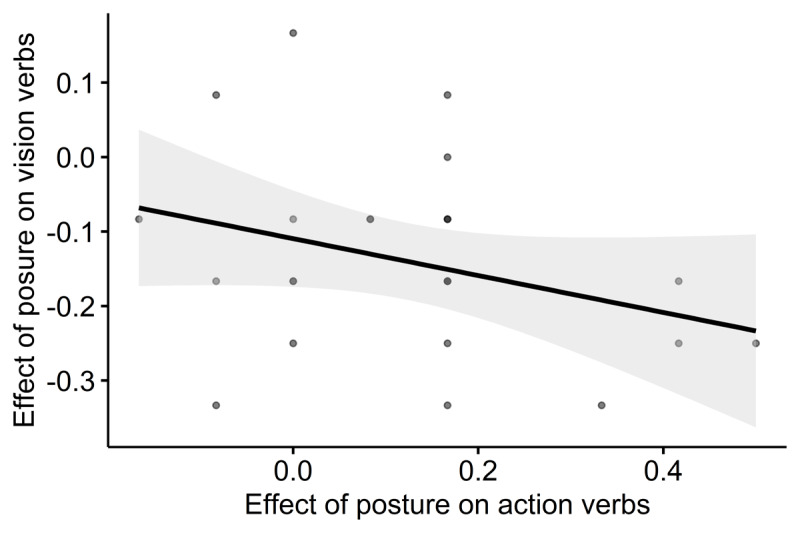
Scatterplot of the correlation between the the individual effects of posture on action and the individual effects of posture on vision phrases in blind participants. Shading represents 95% confidence intervals.

Given these results, one can ask whether a similar mechanism underlies the effects of posture on both action and vision phrases in blind participants. To investigate this question, we further correlated the individual effects of posture on action phrases with the individual effects of posture on vision phrases. The reasoning behind this correlation is that we observed two significant effects of posture in the blind group, specifically in action and vision-related phrases. However, these effects might be driven by different mechanisms. We ran this correlation to explore whether they could instead reflect a shared mechanism. In other words, did blind participants who showed a large postural effect for action phrases also show a large effect for vision phrases? This analysis (see Figure 2) showed a significant correlation in blind participants (*r*(23) = –.34, *p* = 0.047), suggesting that both effects of posture in blind participants arose from a similar mechanism. The correlation for sighted people for sighted participant was not significant (*r*(23) = .06, *p* = 0.760), but the difference between the correlation coefficients of sighted and blind was only close to significance (z = –1.39; *p* = .083).

## Discussion

Theories of embodied and grounded cognition claim that knowledge retrieval is based on the sensorimotor simulation of previous experience (e.g., Barsalou, 1999; Versace et al., 2014). A consequence of this idea is that the grounding of knowledge should be different for people with a different experience of the world, such as blind people. To test this idea, participants were asked to memorize lists of action-related, vision-related, and control phrases while maintaining their hands either at rest in front of them or behind their back. Results showed, first, that the back posture had a similar detrimental effect on the recall performance of action phrases in sighted and blind participants. Second, as expected, posture impacted the memory of vision phrases only in blind participants. Surprisingly, however, the effect was in the opposite direction, with higher recall accuracy in the back posture than in the front posture. Importantly, posture did not affect the recall performance of control phrases.

The evidence that posture affects the recall of vision phrases only in blind participants supports the notion that the representation of vision-related knowledge in blind individuals is, at least partially, remapped onto the motor systems. Indeed, this finding suggests that the bodily state impacted the memory trace of vision phrases only in blind participants. Surprisingly, we observed that blind participants had higher recall accuracy with the hands behind the back, contrary to what was observed with action verbs. If both effects of posture arose from the interference of motor simulation, one might expect that the amplitude of the effect of posture will be similar in a given blind participant. Consistent with this idea, we found that in blind participants, the effect of posture on vision phrases is correlated with the effect of posture on action phrases. Despite their opposite direction, this result suggests a common underlying mechanism, which we hypothesize to be motor simulation.

It is worth noting that although the correlation in the sighted group is far from significance, the difference between the correlation coefficients in the sighted and blind groups does not reach statistical significance, despite their apparent disparity (–.34 in blind and .06 in sighted). However, our main argument rests on the presence of a significant correlation between the significant effect observed in blind participants, which suggests a shared mechanism linking posture effects in action and vision phrases. The absence of a reliable difference between groups likely reflects limited statistical power or variability within the sighted group, where such a mechanism may also be present in a subset of participants. Importantly, this does not undermine our interpretation that a common mechanism could underlie both postural effects observed in blind participants. We suggest two different interpretations that would be consistent with the idea of a common mechanism underlying both effects of posture in blind participants. The first one presupposes a semantic interference phenomenon. Indeed, verbs like “to see”, “to observe”, or “to admire” all involve a similar action component, despite having different meanings. It is possible that they all involve a similar motor simulation, which makes them similar to each other, inducing a semantic interference. We propose that, for blind participants, the hands behind the back inhibited the motor simulation related to vision phrases, which decreased semantic similarity between verbs and alleviated motor semantic interference. This hypothesis is hard to investigate behaviorally since it hinges on the similarity of sensorimotor simulations. However, it makes interesting predictions at the neural level: If this experiment were adapted for fMRI, we could predict that motor/premotor activity associated with verb encoding will be similar, in the blind, for action and vision verbs (supporting the engagement of motor simulations in both cases). However, in the case of vision verbs, it should be much harder to decode the verb identity in those brain regions due to the fact that blind people’s sensorimotor simulations for different visual verbs are highly similar and stereotyped.

Another possibility is that blind participants interpreted the vision-related phrases as being produced by sighted people because this kind of sentence is more typically used by them (Mcginnis, 1981). Consequently, their motoric interpretation of vision phrases must first be inhibited to understand them from the perspective of a sighted person. This would be in line with studies showing that the point of view expressed in language has an impact on motor simulation (Sirigu & Duhamel, 2001). Again, the hands behind the back might have facilitated this inhibition. Even if these interpretations require further investigation, this effect demonstrates, for the first time to our knowledge, the causal involvement of a non-visual modality for the representation of vision-related knowledge in early blind individuals.

This finding might seem at odds with previous research, which has shown that the same visual areas are activated by remappable vision-related concepts in both sighted and blind people, with no difference between the groups (Bi et al., 2016; Peelen & Downing, 2017). One possible explanation for this apparent discrepancy is that these visual areas were actually recycled for other perceptual modalities in early blind individuals (Dormal & Collignon, 2011; Müller et al., 2019) while retaining their functional role (Reich et al., 2011; Striem-Amit & Amedi, 2014). If this is the case, the advantage of a motor interference paradigm such as the one used in the present study is that posture had an effect on motor processing, wherever this motor information is processed in the brain. Another key difference is that the present paradigm might have favored sensorimotor simulation in two different ways. First, a memory task might have enabled a deeper processing than the semantic tasks used in these neuroimaging experiments and thereby promoted sensorimotor simulation (for evidence of the effect of depth of processing on motor simulation, see Glenberg et al., 2008; Papeo et al., 2009; Vukovic et al., 2017). Second, as suggested by previous research (Kiefer, 2019; Moody & Gennari, 2010), using isolated concepts in previous studies may have undermined the sensorimotor simulation process. For example, a verb like “to see” is ambiguous in isolation, as it can be interpreted literally (e.g., “I see a cup”) or figuratively (e.g., “I see a doctor”). The presence of the object in the current study likely disambiguated the verb which promoted sensorimotor simulation.

Another recent study (Bottini et al., 2022) found that the concreteness effect, which is the faster and easier processing of concrete over abstract concepts, is present in blind people for both remappable and non-remappable visual concepts. While this suggests that the concreteness effect does not rely on sensorimotor experience, it does not rule out the involvement of sensorimotor processes in conceptual processing. Indeed, the concreteness advantage might occur at the lexical level, as evidenced by its presence in tasks like lexical decision, and not at a semantic level. This might explain why the remapping of vision-related knowledge does not affect the concreteness effect.

Concerning action verbs, our results confirmed previous studies that have shown that having the hands behind the back has a negative effect on the memory of action-related language (e.g., Dutriaux et al., 2019; Dutriaux & Gyselinck, 2016). This suggests that the memory of action-related knowledge is grounded in the motor system. Additionally, we hypothesized that the memory trace of action phrases would be even more dependent on the action system in the absence of vision. However, analyses did not find any interaction between posture and group. One explanation of this absence of a reliable effect is that the remapping of visual information onto other modalities allowed blind individuals to compensate for the absence of visual information. In any case, our findings indicate that the motor system is causally involved in the memory trace of action-related phrases.

In the context of previous studies (de Vega et al., 2021; Dutriaux et al., 2019; Dutriaux & Gyselinck, 2016), the present results rule out the hypothesis that the effect of posture arose from the presence or absence of the hands in the visual field. However, since posture does not involve any active body movement during the task, one may wonder whether these effects are actually motoric. Evidence shows that the hands behind the back decrease motor excitability during motor imagery (Vargas et al., 2004) and increase beta power in the left frontal gyrus, which has been related to motor inhibition (Donno et al., 2020). More importantly, in a previous study using a similar paradigm, an increase of beta power was observed during the encoding of action phrases but not of other phrases (de Vega et al., 2021; see also Onishi et al., 2022). All these elements provide strong evidence that the hands behind the back inhibit motor activity and indicate that our effects are indeed motoric in nature.

This work aimed to test the sensorimotor grounding of knowledge using two different causal methodologies (Ostarek & Bottini, 2021). First, the differential effect of posture on vision-related phrases in sighted and blind participants showed causally that the early loss of vision impacts the representation of knowledge. Second, the effect of posture indicate that this impact consists in a grounding of vison-related knowledge in the motor system only in blind individuals, while motor-related knowledge is grounded in this system in both sighted and blind individuals. Although further research is needed to understand the unexpected facilitating effect of the hands the back on the memory of action phrases, these findings are the first causal evidence to our knowledge suggesting that the absence of vision leads to a remapping of vision-related knowledge onto other sensorimotor modalities, and open a new window on the representation of knowledge in blind people.

## Data Accessibility Statement

All data and analysis code are available on the Open Science Framework website (https://osf.io/d8h94/).

## Additional File

The additional file for this article can be found as follows:

10.5334/joc.458.s1 Appendix. Lists of stimuli with English translations in italics.
