# Supplementary material for: Memory and Representation of Vision-Related Verbs in Early Blind Individuals
Source: J Cogn. 2025 Aug 22;8(1):45. doi: 10.5334/joc.458 (PMC12372691; doi:10.5334/joc.458)
Supplement: Appendix. — Lists of stimuli with English translations in italics. [file joc-8-1-458-s1.pdf]

Appendix. Lists of stimuli with English translations in italics. For each object and each verb type is indicated the set in which the phrase was assigned to. For instance, in the second row, "Raccogliere un libro" was assigned to set 2.

List	Object	Action Verb	Vision Verb	Control Verb	Action Set	Vision Set	Control Set
1	una melodia<br>*a melody*	Cantare<br>*To sing*	Cantare<br>*To sing*	Cantare<br>*To sing*	filler	filler	filler
	un libro<br>*a book*	Raccogliere<br>*To pick up*	Guardare<br>*To look at*	Adorare<br>*To adore*	2	3	1
	un vassoio<br>*a tray*	Alzare<br>*To lift*	Osservare<br>*To observe*	Volere<br>*To want*	2	3	1
	un telecomando<br>*a remote control*	Spostare<br>*To move*	Individuare<br>*To locate*	Dimenticare<br>*To forget*	1	2	3
	una cintura<br>*a belt*	Posare<br>*To put down*	Adocchiare<br>*To spot*	Apprezzare<br>*To appreciate*	1	2	3
	un pettine<br>*a comb*	Utilizzare<br>*To use*	Trovare<br>*To find*	Preferire<br>*To prefer*	2	3	1
	una tazza<br>*a cup*	Sciacquare<br>*To rinse*	Riconoscere<br>*To recognize*	Apprezzare<br>*To appreciate*	3	1	2
	un asciugamano<br>*a towel*	Afferrare<br>*To grab*	Trovare<br>*To find*	Chiedere<br>*To ask*	3	1	2
	una sigaretta<br>*a cigarette*	Accendere<br>To light	Intravedere<br>To glimpse	Avere<br>To have	1	2	3
	un pacchetto<br>*a package*	Alzare<br>*To lift*	Scorgere<br>*To spot*	Dimenticare<br>*To forget*	3	1	2
2	un problema<br>*a problem*	Capire<br>*To understand*	Capire<br>*To understand*	Capire<br>*To understand*	filler	filler	filler
	una penna<br>*a pen*	Raccogliere<br>*To pick up*	Ammirare<br>*To admire*	Gradire<br>*To like*	3	1	2
	un cellulare<br>*a cellphone*	Accendere<br>*To turn on*	Individuare<br>*To identify*	Udire<br>*To hear*	3	1	2
	una collana<br>*a necklace*	Prendere<br>*To take*	Adocchiare<br>*To spot*	Adorare<br>*To adore*	1	2	3
	una scarpa<br>*a shoe*	Posare<br>*To put down*	Scorgere<br>*Glimpse*	Preferire<br>*To prefer*	1	3	2
	una padella<br>*a pan*	Sciacquare<br>*To rinse*	Vedere<br>*To see*	Preferire<br>*To prefer*	2	3	1
	una tastiera<br>*a keyboard*	Spostare<br>*To move*	Osservare<br>*To observe*	Volere<br>*To want*	3	2	1
	un cucchiaio<br>*a spoon*	Afferrare<br>*To grab*	Individuare<br>*To identify*	Avere<br>*To have*	1	2	3
	un flacone<br>*a perfume bottle*	Svuotare<br>*To empty*	Guardare<br>*To look at*	Volere<br>*To want*	2	1	3
	un portafoglio<br>*a wallet*	Raccogliere<br>*To pick up*	Adocchiare<br>*To spot*	Gradire<br>*To like*	2	3	1

3	una filastrocca<br>*a nursery rhyme*	Cantare<br>*To sing*	Cantare<br>*To sing*	Cantare<br>*To sing*	filler	filler	filler
	uno stuzzicadenti<br>*a toothpick*	Utilizzare<br>*To use*	Intravedere<br>*To glimpse*	Chiedere<br>*To ask*	1	2	3
	un pennarello<br>*a marker*	Spostare<br>*To move*	Individuare<br>*To identify*	Perdere<br>*To lose*	2	3	1
	un cappello<br>*a hat*	Posare<br>*To put down*	Ammirare<br>*To admire*	Adorare<br>*To adore*	2	3	1
	uno zaino<br>*a backpack*	Svuotare<br>*To empty*	Ispezionare<br>*To inspect*	Adorare<br>*To adore*	3	1	2
	un rasoio<br>*a razor*	Afferrare<br>*To grab*	Notare<br>*To notice*	Preferire<br>*To prefer*	3	1	2
	una valigetta<br>*a briefcase*	Alzare<br>*To lift*	Adocchiare<br>*To spot*	Detestare<br>*To detest*	3	1	2
	una lampada<br>*a lamp*	Alzare<br>*To lift*	Osservare<br>*To observe*	Preferire<br>*To prefer*	1	2	3
	un microfono<br>*a microphone*	Stringere<br>*To grip*	Notare<br>*To notice*	Detestare<br>*To detest*	2	3	1
	una chiave<br>*a key*	Raccogliere<br>*To pick up*	Trovare<br>*To find*	Perdere<br>*To lose*	1	2	3
4	una pietra<br>*a stone*	Lanciare<br>*To throw*	Lanciare<br>*To throw*	Lanciare<br>*To throw*	filler	filler	filler
	un gessetto<br>*a piece of chalk*	Stringere<br>*To grip*	Individuare<br>*To identify*	Volere<br>*To want*	1	3	2
	una motosega<br>*a chainsaw*	Accendere<br>*To turn on*	Notare<br>*To notice*	Udire<br>*To hear*	3	1	2
	un anello<br>*a ring*	Posare<br>*To put down*	Ammirare<br>*To admire*	Adorare<br>*To adore*	3	1	2
	un taccuino<br>*a notebook*	Spostare<br>*To move*	Ispezionare<br>*To inspect*	Avere<br>*To have*	2	3	1
	un coltello<br>*a knife*	Sciacquare<br>*To rinse*	Intravedere<br>*To glimpse*	Smarrire<br>*To lose*	2	3	1
	un fiammifero<br>*a match*	Accendere<br>*To light*	Vedere<br>*To see*	Volere<br>*To want*	2	1	3
	una matita<br>*a pencil*	Stringere<br>*To grip*	Riconoscere<br>*To recognize*	Chiedere<br>*To ask*	3	2	1
	una puntina<br>*a pin*	Utilizzare<br>*To use*	Scorgere<br>*To spot*	Smarrire<br>*To lose*	1	2	3
	un vaso<br>*a vase*	Svuotare<br>*To empty*	Ammirare<br>*To admire*	Detestare<br>*To detest*	1	2	3

5	una parola<br>*a word*	Capire<br>*To understand*	Capire<br>*To understand*	Capire<br>*To understand*	filler	filler	filler
	una moneta<br>*a coin*	Raccogliere<br>*To pick u*	Trovare<br>*To find*	Perdere<br>*To lose*	3	1	2
	un orologio<br>*a watch*	Prendere<br>*To take*	Guardare<br>*To look at*	Dimenticare<br>*To forget*	1	2	3
	una sedia<br>*a chair*	Alzare<br>*To lift*	Vedere<br>*To see*	Detestare<br>*To detest*	2	3	1
	un flauto<br>*a flute*	Stringere<br>*To grip*	Ammirare<br>*To admire*	Udire<br>*To hear*	1	2	3
	una spillatrice<br>*a stapler*	Afferrare<br>*To grab*	Notare<br>*To notice*	Avere<br>*To have*	2	3	1
	un righello<br>*a ruler*	Posare<br>*To place*	Intravedere<br>*To glimpse*	Smarrire<br>*To lose*	3	1	2
	una bussola<br>*a compass*	Prendere<br>*To take*	Guardare<br>*To look at*	Dimenticare<br>*To forget*	2	3	1
	una mappa<br>*a map*	Prendere<br>*To take*	Ispezionare<br>*To inspect*	Chiedere<br>*To ask*	3	1	2
	un piatto<br>*a plate*	Sciacquare<br>*To rinse*	Vedere<br>*To see*	Gradire<br>*To like*	1	2	3
6	una sintesi<br>*a summary*	Scrivere<br>*To write*	Scrivere<br>*To write*	Scrivere<br>*To write*	filler	filler	filler
	una chitarra<br>*a guitar*	Stringere<br>*To grip*	Ammirare<br>*To admire*	Udire<br>*To hear*	2	3	1
	un bracciale<br>*a bracelet*	Posare<br>*To put down*	Adocchiare<br>*To spot*	Adorare<br>*To adore*	2	1	3
	una busta<br>*an envelope*	Aprire<br>*To open*	Individuare<br>*To identify*	Gradire<br>*To like*	3	1	2
	un chiodo<br>*a nail*	Prendere<br>*To take*	Intravedere<br>*To glimpse*	Smarrire<br>*To lose*	2	3	1
	un astuccio<br>*a pencil case*	Svuotare<br>*To empty*	Trovare<br>*To find*	Detestare<br>*To detest*	1	2	3
	un biglietto<br>*a ticket*	Prendere<br>*To take*	Riconoscere<br>*To recognize*	Apprezzare<br>*To appreciate*	3	2	1
	un cassetto<br>*a drawer*	Aprire<br>*To open*	Ispezionare<br>*To inspect*	Preferire<br>*To prefer*	1	2	3
	una lampadina<br>*a light bulb*	Accendere<br>*To turn on*	Scorgere<br>*To glimpse*	Volere<br>*To want*	1	3	2
	un bastone<br>*a cane*	Stringere<br>*To grip*	Riconoscere<br>*To recognize*	Avere<br>*To have*	3	1	2

7	una palla<br>*a ball*	Lanciare<br>*To throw*	Lanciare<br>*To throw*	Lanciare<br>*To throw*	filler	filler	filler
	una bottiglia<br>*a bottle*	Sciacquare<br>*To rinse*	Osservare<br>*To observe*	Perdere<br>*To lose*	3	1	2
	un cuscino<br>*a cushion*	Spostare<br>*To move*	Vedere<br>*To see*	Detestare<br>*To detest*	3	1	2
	una racchetta<br>*a racket*	Afferrare<br>*To grab*	Notare<br>*To notice*	Apprezzare<br>*To appreciate*	1	2	3
	un ombrello<br>*an umbrella*	Aprire<br>*To open*	Riconoscere<br>*To recognize*	Dimenticare<br>*To forget*	2	3	1
	una borsa<br>*a bag*	Svuotare<br>*To empty*	Adocchiare<br>*To spot*	Gradire<br>*To like*	2	3	1
	un foglio<br>*a sheet*	Raccogliere<br>*To pick up*	Scorgere<br>*To glimpse*	Chiedere<br>*To ask*	1	2	3
	un rossetto<br>*a lipstick*	Utilizzare<br>*To use*	Scorgere<br>*To glimpse*	Apprezzare<br>*To appreciate*	3	1	2
	un diario<br>*a diary*	Aprire<br>*To open*	Vedere<br>*To see*	Perdere<br>*To lose*	1	2	3
	una radio<br>*a radio*	Accendere<br>*To turn on*	Osservare<br>*To observe*	Udire<br>*To hear*	2	3	1
8	una lettera<br>*a letter*	Scrivere<br>*To write*	Scrivere<br>*To write*	Scrivere<br>*To write*	filler	filler	filler
	una caffettiera<br>*a coffee pot*	Sciacquare<br>*To rinse*	Guardare<br>*To look at*	Udire<br>*To hear*	1	2	3
	una brocca<br>*a pitcher*	Alzare<br>*To lift*	Notare<br>*To notice*	Gradire<br>*To like*	1	2	3
	un ago<br>*a needle*	Utilizzare<br>*To use*	Intravedere<br>*To glimpse*	Smarrire<br>*To lose*	3	1	2
	un apriscatole<br>*a can opener*	Utilizzare<br>*To use*	Trovare<br>*To find*	Chiedere<br>*To ask*	2	3	1
	un accendino<br>*a lighter*	Afferrare<br>*To grab*	Riconoscere<br>*To recognize*	Perdere<br>*To lose*	2	3	1
	un fascicolo<br>*a folder*	Spostare<br>*To move*	Ispezionare<br>*To inspect*	Smarrire<br>*To lose*	1	2	3
	una scatola<br>*a box*	Aprire<br>*To open*	Osservare<br>*To observe*	Dimenticare<br>*To forget*	3	1	2
	un barattolo<br>*a jar*	Svuotare<br>*To empty*	Guardare<br>*To look at*	Avere<br>*To have*	3	1	2
	un armadio<br>*a wardrobe*	Aprire<br>*To open*	Ispezionare<br>*To inspect*	Apprezzare<br>*To appreciate*	2	3	1
